# Prevalence and associated factors of COVID-19 across Italian regions: a secondary analysis from a national survey on physiotherapists

**DOI:** 10.1186/s40945-021-00125-y

**Published:** 2021-12-17

**Authors:** Simone Gambazza, Silvia Bargeri, Isabella Campanini, Roberto Meroni, Andrea Turolla, Greta Castellini, Silvia Gianola, Scientific Technical Committee of AIFI, Simone Gambazza, Silvia Bargeri, Lucia Bertozzi, Isabella Campanini, Davide Cattaneo, Alessandro Chiarotto, Davide Corbetta, Stefania Costi, Roberto Meroni, Susanna Mezzarobba, Tiziano Innocenti, Elisa Pelosin, Maurizio Petrarca, Giacomo Rossettini, Marco Testa, Andrea Turolla, Carla Vanti, Greta Castellini, Silvia Gianola

**Affiliations:** 1 Fondazione IRCCS Ca’ Granda Ospedale Maggiore Policlinico, UOC Direzione delle Professioni Sanitarie, Milano, Via Francesco Sforza 35, 20122 Milan, Italy; 2 IRCCS Istituto Ortopedico Galeazzi, Unit of Clinical Epidemiology, Milan, Italy; 3 LAM-Motion Analysis Laboratory, San Sebastiano Hospital, Correggio, Neuromotor and Rehabilitation Department, AUSL-IRCCS di Reggio Emilia, Reggio Emilia, Italy; 4 Department of Physiotherapy, LUNEX International University of Health, Exercise and Sports, Differdange, Luxembourg; 5 IRCCS San Camillo Hospital, Laboratory of Rehabilitation Technologies, Venice, Italy

**Keywords:** Physiotherapy, Physical therapy, Surveys and questionnaires, Pandemics, Coronavirus, COVID–19, SARS-CoV-2, Coronavirus infections, Disease outbreaks, Prevention & control

## Abstract

**Background:**

Coronavirus disease 2019 (COVID-19) broke out in China in December 2019 and now is a pandemic all around the world. In Italy, Northern regions were hit the hardest during the first wave. We aim to explore the prevalence and the exposure characteristics of physiotherapists (PTs) working in different Italian regions during the first wave of COVID-19.

**Methods:**

Between April and May 2020 a structured anonymous online survey was distributed to all PTs registered in the National Professional Registry to collect prevalence data of a confirmed diagnosis of COVID-19 (i.e., nasopharyngeal swab and/or serological test). A bottom-up agglomerative nesting hierarchical clustering method was applied to identify groups of regions based on response rate. Multivariable logistic regression was used to explore personal and work-related factors associated with a confirmed diagnosis of COVID-19.

**Results:**

A total of 15,566 PTs completed the survey (response rate 43.3%). The majority of respondents (57.7%) were from Northern regions. Considering all respondents, the number of confirmed COVID-19 cases in Northern and Central Italy, was higher compared to those in Southern Italy (6.9% vs. 1.8%, *P* < 0.001); focusing the analysis on respondents who underwent nasopharyngeal swab and/or serological test led to similar findings (14.1% vs. 6.4%, *P* < 0.001). Working in Northern and Central regions was associated with a higher risk of confirmed diagnosis of COVID-19 compared to Southern regions (OR 3.4, 95%CI 2.6 to 4.3). PTs working in Northern and Central regions were more likely to be reallocated to a different unit and changing job tasks, compared to their colleagues working in the Southern regions (10.5% vs 3.7%, *P* < 0.001).

**Conclusions:**

Work-related risk factors were differently distributed between Italian regions at the time of first pandemic wave, and PTs working in the Northern and Central regions were more at risk of a confirmed diagnosis of COVID-19, especially when working in hospitals. Preventive and organizational measures should be applied to harmonize physiotherapy services in the national context.

**Registration:**

https://osf.io/x7cha

**Supplementary Information:**

The online version contains supplementary material available at 10.1186/s40945-021-00125-y.

## Key messages

**What’s already known about this topic:**

- During the first wave of COVID-19, more than 10% of the positive cases in Italy were healthcare workers (HCWs).
- The rapid spread of the virus has affected all HCWs, including physiotherapists (PTs) who played a central role in the management of COVID-19 patients.

**What does the study add?**

- PTs working in the Northern and Central regions were more at risk of a confirmed diagnosis of COVID-19 during the first wave of the COVID-19 pandemic.
- In case of widespread infectious diseases, contingency plans where PTs are reallocated to different units and tasks should embody proper preventive and organizational measures to reduce infection risks among HCWs.

## Introduction

On January 31, Italy declared a public health emergency when two Chinese tourists in Rome tested positive for the new *severe acute respiratory syndrome-related coronavirus 2* (SARS-CoV-2), addressed by the World Health Organization on March 11, 2020 as a public health emergency of international concern [1]. In February, 11 municipalities in Northern Italy, located in Lombardy and Veneto respectively, were placed under quarantine after being identified as the centers of the two primary Italian outbreaks. On March 8, 2020, the Italian Government extended the quarantine to the entire Lombardy region and to 14 other Northern provinces. The following day a nationwide lockdown was declared, resulting in more than 60 million people confined to their homes. Travels, non-essential industrial activities and social interactions were banned to contain the spread of the virus and the potentially devastating consequences of the coronavirus disease 2019 (COVID-19) [2, 3]. The national lockdown ended on May 18, 2020 [4]. However, while people had to stay at home to reduce the spread of this virus, health-care workers (HCWs) have been working, exposed to a higher risk of infection [5], especially during the early months. By June 22, 2020 Italy recorded 29,282 HCWs infected, accounting for the 12% of total COVID-19 cases in the country [6]. Among HCWs, the virus’s rapid spread has also upset the physiotherapists (PTs) [7]. As incidence of COVID-19 increased, many countries have considerably diverted healthcare resources converting the rehabilitation departments into COVID-19 units [8], and reducing outpatient rehabilitation treatments [9] in response to social distancing policies implemented and to minimize the spread of the infection through the population.

Physiotherapists provide frontline care during COVID-19 disease, having a relevant role in managing patients admitted to hospital with acute COVID-19 [10] and promoting functional recovery after the acute phase of respiratory distress [9, 11]. In addition, physiotherapists continuously maintain essential rehabilitation services across the care continuum (e.g., in case of trauma or stroke) [12].

During the first wave, among Italian PTs who underwent tests to confirm the diagnosis almost 1 out of 7 were COVID-19 positive [13]. However, prevalence across Italian regions can be connoted by remarkable differences. The aim of our study was to inspect the prevalence and the exposure characteristics of PTs working in different Italian regions during the first wave of the COVID-19 pandemic.

## Methods

### Study design

This is a secondary analysis of a national structured online closed survey on PTs [13]. Prevalence data describing the picture at the national level are published elsewhere [13]. This study complies with the Declaration of Helsinki and good clinical practices. We generalized sensitive data in order to ensure an anonymized dataset and comply with data protection regulations. European advisory body and European Commission in this pandemic did not require ethical approval [14, 15]. We followed the available guidelines for the reporting of Survey-Based Research [16, 17] (Additional file 1) according to the STROBE statement [18].

### Survey questionnaire

We used SurveyMonkey platform [19] to pilot and develop a web-based questionnaire, which was available for 4 weeks, starting from April 28, 2020. The questionnaire was built and piloted by all the members of the Scientific Committee of Associazione Italiana Fisioterapia (AIFI) and then distributed to all PTs registered in Italy within the national professional registry. The different sections included questions related to: (I) “demographic characteristics”, (II) “personal risk of exposure”, (III) “work-related risk of exposure” and (IV) “prevalence of COVID-19”. The questionnaire was formulated in Italian (https://osf.io/x7cha) and reported in English in Additional file 2.

The informed consent was assumed when respondents completed and submitted the survey after reading the purpose statement of the study, the anticipated time needed to complete the survey (5 min) and the privacy about the data collection. Responses were treated anonymously. Further details about the management of the survey (e.g., multiple responses, id linked to IP address) are already reported in the previously published study [13].

### Statistical analyses

Metrics are expressed as mean and standard deviation (SD), median and interquartile range (IQR) or absolute value and frequency, summarized in tables and figures. For the regional response rate, we stratified by the 20 regions all members registered in the Italian National Professional Registry (Federazione Nazionale Ordini dei Tecnici Sanitari di Radiologia Medica, delle Professioni Sanitarie Tecniche, della Riabilitazione e della Prevenzione) which provides aggregated comulative data at regional level [20, 21]..

A bottom-up agglomerative nesting hierarchical clustering method based on Ward’s criterion was applied to identify Italian regions based on homogeneous response rates. The goodness of clustering was assessed by silhouette and agglomerative coefficients [22]. The optimal number of clusters was chosen according to the gap statistic method using 500 bootstrapped samples.

In this study a confirmed diagnosis of COVID-19 was defined in case the participant reported a positive nasopharyngeal swab (NPS) and/or serological test, since there was a paucity of available diagnostics during the first wave of pandemic [23]. Using logistic regression we investigated whether a confirmed diagnosis of COVID-19 (dependent variable) was associated with the identified clusters, once controlling for few other covariates: personal factors (age, sex and presence of comorbidities), the working facility and the professional field. Further adjusting covariates were changing job task and being reallocated to a different unit. Akaike information criterion [24, 25] was used as a guide for model selection, which did not include interactions.

Under the assumption of missingness at random, 50 multiple imputations using a non-parametric approach (van Buuren’s Type I matching) in conjunction with bootstrap to incorporate all uncertainties was used to reduce bias in logistic regression estimates and substantial loss in sample size [26], due to the extent of missing data in the selected covariates. Inference on considered parameters was obtained by combining estimates over 50 imputed data sets using Rubin’s rules. Sensitivity analysis was then reported to assess the plausibility of the estimates over complete case analysis.

Two-sample test of proportions was used to assess differences between clusters, together with 95% confidence intervals (CIs). All statistical tests were two-sided and *P*-value < .05 was considered statistically significant. All analyses were performed using R Core Team [27], version 3.6.2., with *factoextra and Hmisc* packages added.

## Results

### Response rate and cluster analysis

**Fig. 1:**
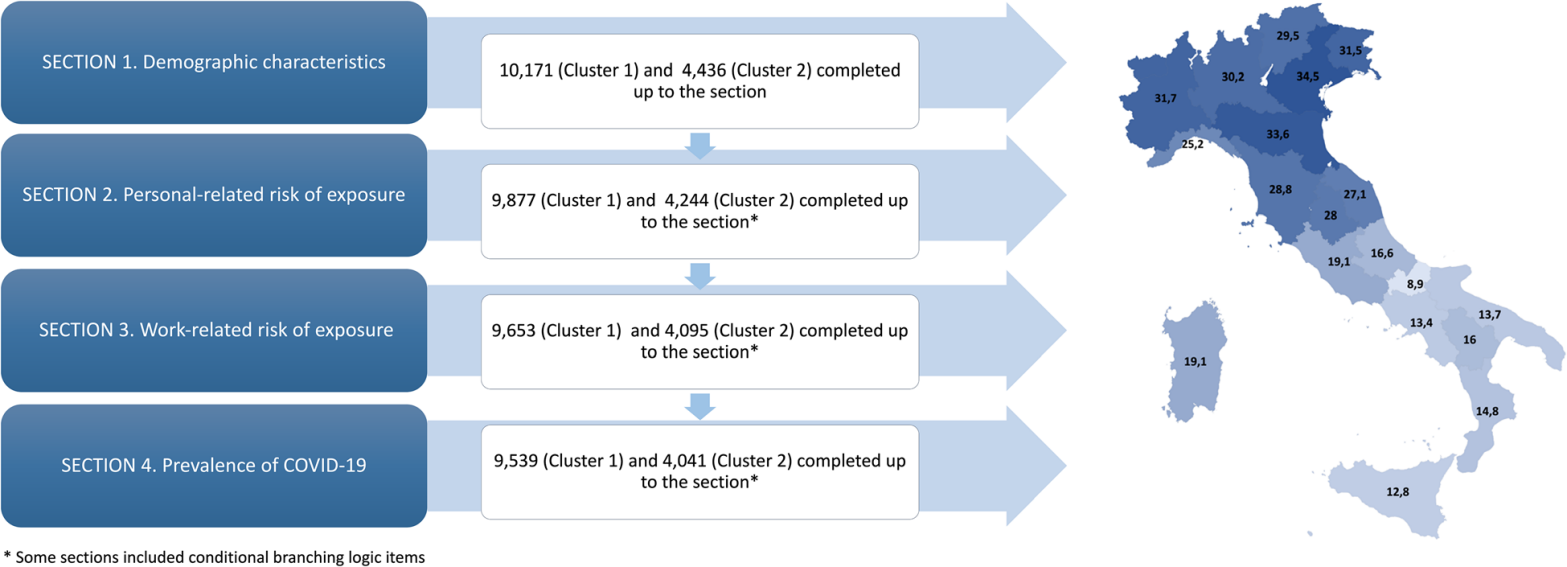
Flow diagram of participants in each section based on cluster analysis and response rate of each region. A gradient map of Italy shows higher response rate in dark blue and lower response rate in light blu

Overall, we had 15,566 respondents out of 35,938 active members, yielding a response rate of 43.3%. Through cluster analysis, we identified two groups of regions in Italy: Cluster 1 is made of 10,171 PTs from the Northern and Central Italian regions (Piedmont and Aosta Valley, Liguria, Lombardy, Veneto, Friuli-Venezia-Giulia, Trentino-Alto-Adige, Emilia-Romagna, Tuscany, Marche and Umbria), whereas Cluster 2 consists of 4436 PTs from Southern Italy and Islands (Abruzzo, Lazio, Molise, Campania, Apulia, Basilicata, Calabria, Sicily and Sardinia). Additional details about cluster analysis are reported in Additional file 3. For all the questionnaire sections, the flow diagram of the two clusters and the response rates of every region is reported on a gradient map (Fig. 1).

### Respondents’ characteristics

The median age of all respondents was 40 years old (IQR 32–50) and 63% were women. Overall, 57.7% of participants were from Northern regions, 23.2% were from Central regions and 19.1% from Southern and Island regions. Further details about respondents’ characteristics are reported in the previously published study [13].

### Prevalence of COVID-19

We analyzed the prevalence of confirmed COVID-19 diagnosis (NPS and/or serological test) in the two clusters. The confirmed COVID-19 cases on all respondents in each cluster were 5.1% higher (95%CI: 4.5 to 5.8%) in Northern-Central than Southern regions, respectively 6.9% vs. 1.8%. Analogously, the confirmed COVID-19 cases on those who underwent tests (i.e., NPS and/or serological test) were 7.7% higher (95%CI: 5.9 to 9.4%) in Cluster 1 compared to Cluster 2, 14.1% vs. 6.4% respectively. Analyzing data at a regional level, we found that Veneto performed the highest number of NPSs (44.5%), whereas Apulia the lowest (9.8%) (Additional file 4, Fig. S1). Considering as denominator the number of respondents per each region, Trentino-Alto-Adige had the highest rate of positive cases (7.4%), followed by Piedmont & Aosta Valley (6.4%) and Lombardy (5.5%). In contrast, Southern regions showed the lowest (Additional file 4, Fig. S2). Considering as denominator the NPSs performed per each region, Liguria had the highest rate of positive cases (22.6%), whereas Southern regions the lowest (Additional file 4, Fig. S3). The top five symptoms reported were similar between Northern-Central and Southern regions (fatigue and tiredness: 67.6% vs 81.8%, respectively), loss of smell (64.2%% vs 67.3%, respectively), aches and pains (59.6% vs 70.9%, respectively), loss of taste (57.5% vs 65.5%, respectively) and headache (49.3% vs 67.3%, respectively). Further details are provided in the online Additional file 5.

### Work-related characteristics of Italian PTs across regions

**Fig. 2:**
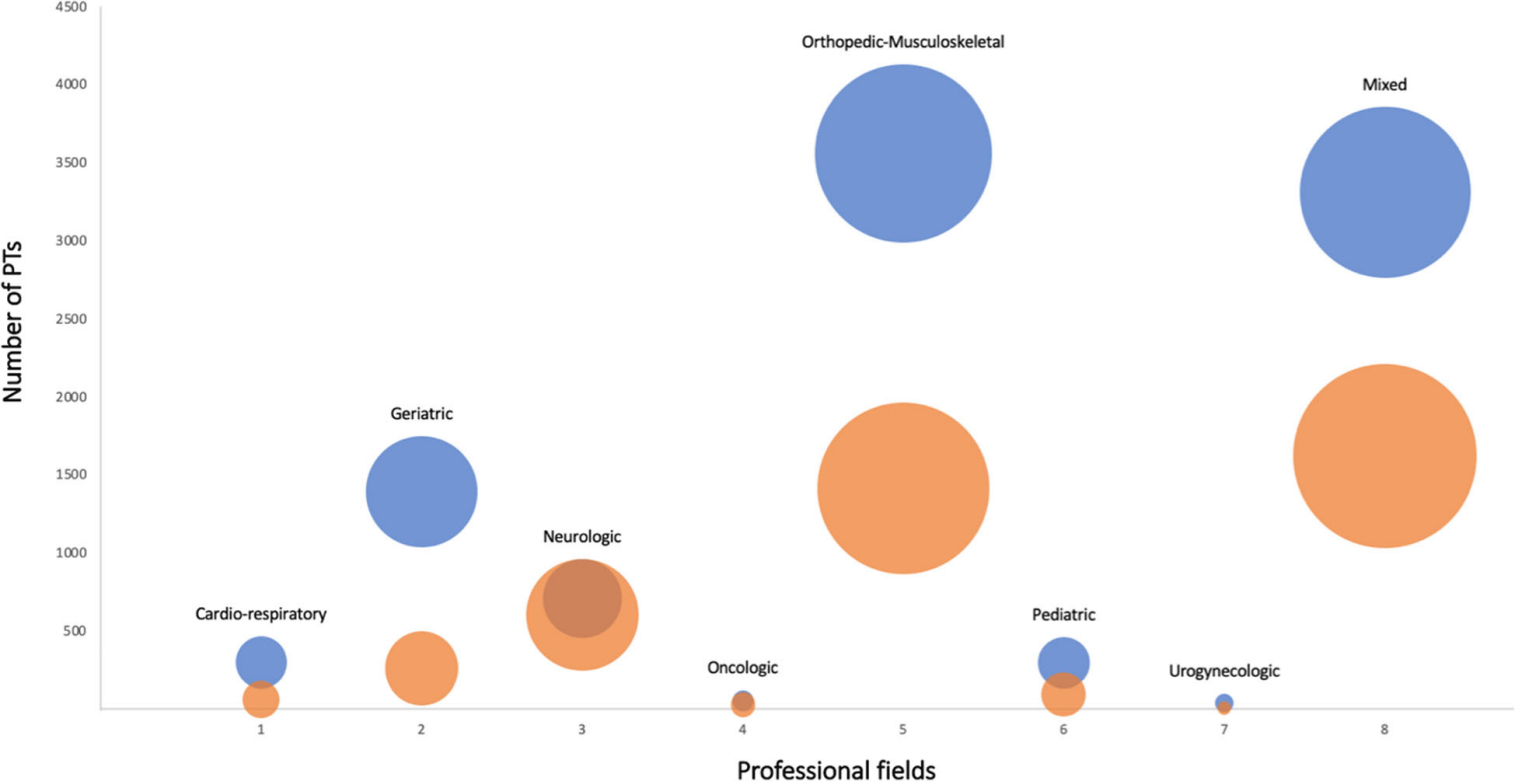
Distribution of PTs and their professional fields between clusters. Colors denote Northern-Central (blue) and Southern Italian regions (orange). Size of nodes are proportional to the number of the PTs working in the corresponding field within the own cluster. Cluster 1 = Piedmont and Aosta Valley, Liguria, Lombardy, Veneto, Friuli-Venezia-Giulia, Trentino-Alto-Adige, Emilia-Romagna, Tuscany, Marche and Umbria; Cluster 2 = Abruzzo, Lazio, Molise, Campania, Apulia, Basilicata, Calabria, Sicily and Sardinia

We found statistically significant differences between Northern-Central and Southern regions in the distribution of work-related characteristics of respondents. More PTs in Cluster 1 were called to be on duty (70.7% Cluster 1 vs 58.3% Cluster 2, *P* < 0.001) whereas in Cluster 2 we found higher rate of PTs not on duty (22.9% Cluster 1 vs 36.1% Cluster 2, *P* < 0.001) due to professional activity suspended by law because of COVID-19 outbreak or temporary lay-off. In Cluster 1 more PTs worked in public healthcare institutions (e.g., hospitals) (70.1% Cluster 1 vs 59.7% Cluster 2, *P* < 0.001) and were usually employed in the musculoskeletal field (36.9% Cluster 1 vs 34.5% Cluster 2, *P* < 0.001). Working in cardio-respiratory field showed the lowest rate in Cluster 2 compared to Cluster 1 (3.1% Cluster 1 vs 1.6% Cluster 2, *P* < 0.001) (Fig. 2). In Cluster 1 more PTs were reallocated to a different unit and changed job related tasks (10.5% Cluster 1 vs 3.7% Cluster 2, *P* < 0.001). In addition, more PTs in Cluster 1 answered that SARS-CoV-2 infection occurred by contact with positive patients without Personal Protective Equipment (PPE) (31.5% Cluster 1 vs 22.9% Cluster 2, *P* < 0.05) whereas in Cluster 2 more PTs answered that they were infected without contact with positive patients (41.9% Cluster 1, 50.7% Cluster 2, *P* < 0.05). Further details are reported in Table 1.

**Table 1:** Work-related characteristics of respondents between clusters

Work-related characteristics					
	Cluster 1 (*N* = 9653)		Cluster 2 (*N* = 4095)		*P*-value
**PROFESSIONAL FIELD**					
Cardio-Respiratory	299	3.1	64	1.6	< 0.001
Geriatric	1394	14.4	260	6.3	< 0.001
Neurologic	707	7.3	604	14.7	< 0.001
Oncologic	53	0.5	29	0.7	0.2678
Orthopedic-Musculoskeletal	3558	36.9	1413	34.5	< 0.01
Pediatric	295	3.1	94	2.3	< 0.05
Urogynecologic	39	0.4	9	0.2	0.094
Mixed	3308	34.3	1622	39.6	< 0.001
**WORKING FACILITIES**					
Residential care home	1241	12.9	353	8.6	< 0.001
Private/public rehabilitation clinics	1882	19.5	1159	28.3	< 0.001
More than one (e,g., residential care home + private setting)	1222	12.7	414	10.1	< 0.001
Private/public hospital	2413	25	520	12.7	< 0.001
Private setting	2343	24.3	806	19.7	< 0.001
Home	552	5.7	843	20.6	< 0.001
**CURRENT EMPLOYMENT STATUS**					
On Duty At The Workplace	6827	70.7	2388	58.3	< 0.001
On duty (tele-work)	288	3	171	4.2	< 0.001
Not on duty (on vacation, leave, parental leave, layoff, sick leave)	951	9.9	743	18.1	< 0.001
Not on duty (professional activity suspended by law or suspended due to covid-19 outbreak)	1255	13	739	18	< 0.001
Not on duty (diagnosis of covid-19)	247	2.6	33	0.8	< 0.001
Quarantined (suspected covid-19)	85	0.9	21	0.5	< 0.05
**LIVING WITH SOMEONE INFECTED**					
Living With Someone Infected	598	6.1	255	6.0	0.9162
Using a separate room	299	49.8	119	47.2	0.5004
**REORGANIZATION AT THE WORKPLACE**					
Reallocation to different unit	719	10.5	89	3.7	< 0.001
Changing job task	727	10.7	55	2.3	< 0.001
**CONTACT WITH POSITIVE PATIENTS**					
With PPE	389	26.6	37	26.4	0.9524
Without PPE	459	31.5	32	22.9	< 0.05
With or without PPE	848	58.1	69	49.3	< 0.05
No contact with positive patients	611	41.9	71	50.7	< 0.05

Data are presented as count and percentage (%)

Custer 1 = Piedmont and Aosta Valley, Liguria, Lombardy, Veneto, Friuli-Venezia-Giulia, Trentino-Alto-Adige, Emilia-Romagna, Tuscany, Marche and Umbria; Cluster 2 = Abruzzo, Lazio, Molise, Campania, Apulia, Basilicata, Calabria, Sicily and Sardinia

*PPE* Personal Protective Equipment

### Personal and work-related factors associated with COVID-19

We found evidence of association between clusters and the probability of testing COVID-19 positive, which was lower for PTs in Southern regions. Overall, working in Northern and Central respect to Southern Italian regions increased the log odds of testing COVID-19 by 1.21 (OR 3.4, 95%CI: 2.6 to 4.3).

The I and III quartiles of age were 32 and 50 years, respectively, so the half-sample odds ratio for age was 1.17 (95%CI:1.02 to 1.33). Female sex showed a protective effect towards the probability of testing COVID-19 positive (OR 0.68, 95%CI:0.57 to 0.80). Further evidence of association came with the professional field of PTs and their working facilities. Particularly, if compared to the cardio-respiratory professional field, all other fields showed a protective effect over the probability of testing positive. However, statistical significance was achieved only in the orthopedic-musculoskeletal field (OR 0.47, 95%CI:0.31 to 0.72). Compared to hospital setting, working in private/public rehabilitation clinics, at home or in the private setting decreased the log odds by 0.35, 1.23 and 1.69, respectively (Table 2).

**Table 2:** Logistic regression estimates after complete case analysis and multiple imputation

	Complete-case analysis (*N* = 9164)			Multiple imputation (*N* = 14,607)		
	Coefficient	SE	*P*-value	Coefficient	SE	*P*-value
**Cluster 2 versus Cluster 1**	−1.26	0.19	< 0.0001	− 1.22	0.13	< 0.0001
**Males versus females**	0.38	0.12	0.0009	0.39	0.08	0.0202
**Age, yrs**	0.014	0.005	0.0026	0.009	0.004	0.0257
**At least 1 comorbidities versus none**	0.18	0.12	0.1205	0.12	0.08	0.1754
**Professional field**			0.0005			< 0.0001
(Cardio-respiratory as reference)	–	–	–	–	–	–
Geriatric	−0.43	0.32	0.3881	−0.03	0.23	0.9111
Neurologic	−0.26	0.3	0.0105	−0.29	0.23	0.2017
Orthopedic-Musculoskeletal	−0.72	0.28	0.1041	−0.75	0.22	0.0006
Urogynecologic and Oncologic	−1.68	1.04	0.7709	−0.29	0.43	0.5095
Pediatric	−0.11	0.38	0.9873	−0.25	0.29	0.3921
Mixed	−0.004	0.25	0.9158	−0.032	0.2	0.8694
**Working Facilities**			< 0.0001			< 0.0001
(Private/public hospitals as reference)	–	–	–	–	–	–
Residential Care Home	0.02	0.22	0.9158	0.22	0.15	0.1472
Private/public rehabilitation clinics	−0.41	0.17	0.0137	−0.35	0.12	0.0037
Home	−1.55	0.4	< 0.0001	−1.23	0.25	< 0.0001
Mixed	0.44	0.16	0.0047	0.17	0.12	0.1748
Private setting	−1.47	0.27	< 0.0001	−1.69	0.21	< 0.0001
**Reallocation to a different unit (Yes versus No)**	0.27	0.16	0.0862	0.26	0.16	0.1078
**Changing job task (Yes versus No)**	0.21	0.16	0.1794	0.29	0.16	0.0746
**Constant**	−3.3	0.32	< 0.0001	−2.7	0.25	< 0.0001

Estimates are expressed as log odds

Cluster 1 = Piedmont and Aosta Valley, Liguria, Lombardy, Veneto, Friuli-Venezia-Giulia, Trentino-Alto-Adige, Emilia-Romagna, Tuscany, Marche and Umbria; Cluster 2 = Abruzzo, Lazio, Molise, Campania, Apulia, Basilicata, Calabria, Sicily and Sardinia

*SE* Standard Error

After the considered adjustments, 30% odds increased remained when PTs changed their job tasks (i.e., OR 1.34, 95%CI:0.97 to 1.85) or were reallocated to a different unit (i.e., OR 1.30, 95%CI: 0.94 to 1.80); however, every hypothesis from no association up to 80% odds increase had a *P* > 0. 05.

Characteristics of PTs with missing COVID-19 tests are reported in Additional file 6, Table S1. Generally, the pattern of missingness both involved changing job task and being reallocated to a different unit. Taken alone, no key predictors of such missing pattern were identified. The imputed model showed consistent estimates and lower standard errors compared to the complete-case analysis. Although validating data for prospective use was not necessary, multiple imputation reduced the overfitting by 3.4%, showing a shrinkage estimate of 0.97, also indicating that this model will validate on new data about 2.8% worse than on this dataset.

## Discussion

In this survey, PTs working in Northern and Central regions were found to have greater chances of being positive than those in Southern regions and Islands. Indeed, higher prevalence of COVID-19 cases was reported by PTs of Northern and Central regions of Italy. Similar findings in all HCWs are reported in the national bulletin [28] as well as in published studies [29, 30]. In the Northern and Central regions the infection rate of PTs was 11 times higher than that reported in the general population according to the regional data updated on June 32,020, which showed 207,636 cumulative cases [28] (33,691,608 units [31]) with a prevalence of 0.6% [32]. This can be explained by the prompt lockdown, when the Italian Government closed all non-essential businesses and industries, and restricted the movement of people across all country preventing the COVID-19 epidemic in the Southern regions to rise to the high levels that were already occurring in the North [3]. In fact, on May 31, national data in Italy showed that the most strongly affected regions were those of Northern and Central: Lombardy, Piedmont and Aosta Valley, Emilia Romagna, Veneto and Tuscany [33]. Among Italian government measures, we found that Veneto was the most active region in performing NPS tests, whereas Apulia was the least, probably due to some different preventive and control strategies between regions (e.g., lockdown, contact tracing and NPS tests) [34, 35].

Furthermore, analyzing the work-related variables of PTs in both clusters, we found that more PTs in Northern and Central regions worked in healthcare institutions, and during the pandemic more PTs of Cluster 1 were re-allocated to a different unit and changed job task respect to Cluster 2. These findings should also be interpreted taking into account the early stages of SARS-CoV-2 pandemic in Italy, which caused a crisis in the standards of care of the National Health System, requiring significant resources to be diverted due to the large number of critically ill patients [36, 37]. The reorganization of health care facilities, therefore, was part of a larger plan extended to the entire national, regional and local health services, in particular in the Northern regions, to face the emergency [38]. Consistently, we found that more PTs in Cluster 1 answered that they were infected through contact with positive patients without wearing PPE, whereas in Cluster 2 more PTs answered that infection occurred for other causes. On one hand we should take into account the delay in the implementation of routine prevention and control measures for all HCWs at the time of first wave in Italy, including the lack of preventive equipment (i.e., PPE) as well as the reluctance to their use [39]. The lack of agreement among healthcare organizations regarding whether surgical masks or N95 respirators were effective did not help in the first wave of pandemic [40–43]. On the other hand, PTs of Cluster 2 may have been infected in the community. In fact, PTs from Southern regions were less exposed in the early stage of the pandemic with almost half of them not being working (many activities were suspended by law or suspended due to COVID-19 outbreak or they were temporarily laid off). This hypothesis is in line with a Dutch cross-sectional study [44] that found that during the early phase of local spread, a substantial proportion of HCWs with self-reported fever or respiratory symptoms were likely the consequence of community acquired infections.

Finally, looking at the association between some considered variables and the probability of testing COVID-19 positive, we found that female sex and younger age turned to be protective factors, as well as working in the orthopedic field compared to treating patients affected mainly by cardio-respiratory diseases. Age and sex have been already reported as key features associated with a lower chance of a COVID-19 diagnosis [45]. Current evidence suggests that a person can be infected when aerosols or droplets containing the virus are inhaled or come directly into contact with the eyes, nose or mouth [46, 47]. PTs working in the cardio-respiratory field were likely at higher risk of being infected, considering that airway clearance techniques and the use of respiratory equipment are part of their main activities [48]. Furthermore, considering the imprecise but existent effect on the chance to be COVID-19 positive, quick solution to displace personnel from one unit to another or to change their job related tasks should be carefully pondered in future scenarios by healthcare managers [49]. Altogether, these findings should be considered in future policies, recognizing different personal [13] and work-associated risk to PTs, when exposed to the broad and heterogenous fields of delivered care. Public health policy should continue to reinforce the principles to early identify suspected COVID-19 cases or other risks and to invest efforts in staff training and education (e.g., procedures in case of contact with positive patients), workplace culture, organizational leadership and management in order to help policy reducing the impact of COVID-19 among HCWs [50].

### Strength and limitations

Several limitations should be acknowledged. Southern Italy had the lowest response rate that could be influenced by the low prevalence of disease at the time of the first COVID-19 wave. In fact, some participants could have been encouraged to complete the survey only because they experienced the disease. However, this rate is coherent with other findings during the first Italian wave of pandemic [51].

We reported the prevalence of positive cases per region calculated with respect to both the total number of participants and the total number of participants who underwent NPS tests; however, this information should be interpreted with caution because we did not standardize the data regarding the overall number of PTs and the number of PTs who underwent NPS tests per region. As in all the cross-sectional studies, the prevalence of estimates can be biased by information and selection biases. Overall, these findings were collected via a self-reported survey limiting the accuracy of the estimates. Despite these limitations, our study is characterized by several unique strengths. We enrolled 15,566 respondents to understand and measure the burden of disease among PTs in Italy. We analyzed the data by the means of unsupervised clustering algorithm, based on the response rate only, which might directly reflect national regional imbalance at the time of COVID-19 first wave. The cluster with the most significant statistical power included 70% of the whole sample of respondents, all of whom were from the Northern and the Central regions of Italy. We used multivariable logistic regression to adjust for important personal and work-related factors, and we choose multiple imputation to present unbiased estimates. We have reported for the first time regional differences in the practice of physiotherapy related to a global public emergency.

### Implication and conclusions

Our findings suggest that during the first wave of COVID-19 in Italy, work-related factors in Northern-Central regions were associated with higher odds of a confirmed diagnosis of COVID-19 in PTs. Health care institutions are expected to ensure a more effective and timely response to future pandemics. The National Health System should harmonize prevention and control strategies all over the Italian territory (e.g., NPS test provided, use of PPE) avoiding inequity among regions, possibly due to different proportion of confirmed diagnosis between regions. Health care institutions in all regions should be promptly organized for upcoming emergencies, being aware that PTs employed in public/private hospitals might deserve more attention. It is advisable and desirable that decision-makers avoid changing the tasks PTs engage in and reallocating them to new units requiring different skills and competencies (e.g., from orthopedic-musculoskeletal to respiratory settings) unless it is strictly necessary. Promoting expertise into the respiratory care and reorganizing wards with an adequate number of skilled PTs (i.e., PTs specialized in cardiorespiratory physiotherapy) should be of priority, in particular when reallocation is inevitable. Finally, since the current evidence demonstrates that the implementation of prevention and control measures (e.g., wearing N95 masks as PPE) can prevent the infection, adequate supplies of PPEs should be always guaranteed.

## Supplementary Information

**Additional file 1.** Checklist for Reporting Results of Internet E-Surveys (CHERRIES).

**Additional file 2.** Questionnaire.

**Additional file 3.** Cluster analysis.

**Additional file 4.** Additional analyses Prevalence of COVID-19 in all regions.

**Additional file 5.** Additional analyses symptoms of COVID-19 in all regions.

**Additional file 6.** Additional analyses risk exposure of COVID-19 in all regions.

## Data Availability

All data generated or analyzed during this study are included in this published article [and its additional files]. Row data are stored in an open platform at the following link: https://osf.io/x7cha.

## Abbreviations

AIFI: Associazione Italiana Fisioterapia
COVID-19: Coronavirus disease 2019
HCW: Health Care Workers
NPS: Naso Pharyngeal Swabs
OR: Odds ratio
PPE: Personal Protective Equipment
PTs: Physiotherapists
SARS-COV-2: Severe acute respiratory syndrome coronavirus 2

## References

[1] WHO. Director-General’s opening remarks at the media briefing on COVID-19-11 March 2020. World Health Organization; 2020. Available from: https://www.who.int/dg/speeches/detail/who-director-general-s-opening-remarks-at-the-media-briefing-on-covid-19%2D%2D-11-march-2020. Accessed on 29 Mar 2020.

[2] Remuzzi A, Remuzzi G (2020). COVID-19 and Italy: what next?. Lancet..

[3] Sebastiani G, Massa M, Riboli E (2020). Covid-19 epidemic in Italy: evolution, projections and impact of government measures. Eur J Epidemiol.

[4] https://it.wikipedia.org/wiki/Gestione_della_pandemia_di_COVID-19_in_Italia. Accessed on Jan 2021.

[5] The L (2020). COVID-19: protecting health-care workers. Lancet..

[6] Epicentro ISS. https://www.epicentro.iss.it/coronavirus/bollettino/Infografica_22giugno%20ITA.pdf. Accessed on 30 June 2020.

[7] ISS R (2020). Aggiornamento nazionale, Epidemia COVID-19.

[8] Brugliera L, Spina A, Castellazzi P, Cimino P, Tettamanti A, Houdayer E (2020). Rehabilitation of COVID-19 patients. J Rehabil Med.

[9] Carda S, Invernizzi M, Bavikatte G, Bensmail D, Bianchi F, Deltombe T (2020). COVID-19 pandemic. What should physical and rehabilitation medicine specialists do? A clinician’s perspective. Eur J Phys Rehabil Med.

[10] Battaglini D, Robba C, Caiffa S, Ball L, Brunetti I, Loconte M, Giacobbe DR, Vena A, Patroniti N, Bassetti M, Torres A, Rocco PRM, Pelosi P (2020). Chest physiotherapy: an important adjuvant in critically ill mechanically ventilated patients with COVID-19. Respir Physiol Neurobiol.

[11] Thomas P, Baldwin C, Bissett B, Boden I, Gosselink R, Granger CL, Hodgson C, Jones AYM, Kho ME, Moses R, Ntoumenopoulos G, Parry SM, Patman S, van der Lee L (2020). Physiotherapy management for COVID-19 in the acute hospital setting: clinical practice recommendations. J Phys.

[12] Prvu Bettger J, Thoumi A, Marquevich V, De Groote W, Rizzo Battistella L, Imamura M, et al. COVID-19: maintaining essential rehabilitation services across the care continuum. BMJ Glob Health. 2020;5(5) PubMed PMID: 32376777. 10.1136/bmjgh-2020-002670 PMC7228480 32376777

[13] Gianola S, Bargeri S, Campanini I, Corbetta D, Gambazza S, Innocenti T, et al. The spread of COVID-19 among 15,000 physical therapists in Italy: a cross-sectional study. Phys Ther. 2021;8(8). 10.1093/ptj/pzab123 PubMed PMID: 33970270. Epub 2021/05/11. eng. 10.1093/ptj/pzab123 PMC8136025 33970270

[14] Commission E. 2020. https://ec.europa.eu/research/participants/data/ref/h2020/grants_manual/hi/ethics/h2020_hi_ethics-data-protection_en.pdf. Accessed on 19 May 2020.

[15] PARTY ADPW. Anonymisation techniques. https://ec.europa.eu/justice/article-29/documentation/opinion-recommendation/files/2014/wp216_en.pdf. Accessed on Nov 2020.

[16] Bennett C, Khangura S, Brehaut JC, Graham ID, Moher D, Potter BK, Grimshaw JM (2010). Reporting guidelines for survey research: an analysis of published guidance and reporting practices. PLoS Med.

[17] Eysenbach G (2004). Improving the quality of web surveys: the checklist for reporting results of internet E-surveys (CHERRIES). J Med Internet Res.

[18] von Elm E, Altman DG, Egger M, Pocock SJ, Gotzsche PC, Vandenbroucke JP (2007). The strengthening the reporting of observational studies in epidemiology (STROBE) statement: guidelines for reporting observational studies. PLoS Med.

[19] SurveyMonkey. https://it.surveymonkey.com/. Accessed on 20 Apr 2020.

[20] TSRM-PSTRP F. FNO TSRM-PSTRP - Ricerca Iscritti. 2020. Available from: https://webiscritti.tsrmweb.it/Public/RicercaIscritti.aspx. 27 Mar 2020.

[21] Wikipedia (2020). First-level NUTS of the European Union.

[22] Langfelder P, Horvath S. Fast R functions for robust correlations and hierarchical clustering. J Stat Softw. 2012;46(11) PubMed PMID: 23050260. Pubmed Central PMCID: PMC3465711. eng. PMC3465711 23050260

[23] Peeling RW, Wedderburn CJ, Garcia PJ, Boeras D, Fongwen N, Nkengasong J, Sall A, Tanuri A, Heymann DL (2020). Serology testing in the COVID-19 pandemic response. Lancet Infect Dis.

[24] Burnham KP, Anderson DR. Model Selection and Multimodel Inference: A Practical Information-Theoretic Approach. New York: Springer; 2002.

[25] Akaike H (1974). A new look at the statistical model identification. IEEE Trans Autom Control.

[26] Carpenter JR, Smuk M. Missing data: A statistical framework for practice. Biom J. 2021;63(5):915–947. 10.1002/bimj.202000196. Epub 2021 Feb 24. PMID: 33624862. 10.1002/bimj.202000196 PMC7615108 33624862

[27] R Core Team (2019). R: a language and environment for statistical computing.

[28] Istituto Superiore di Sanità, EPIDEMIA COVID-19 Aggiornamento nazionale 3 giugno 2020. https://www.epicentro.iss.it/coronavirus/bollettino/Infografica_3giugno%20ITA.pdf. Accessed on Mar 2021.

[29] Fusco FM, Pisaturo M, Iodice V, Bellopede R, Tambaro O, Parrella G, di Flumeri G, Viglietti R, Pisapia R, Carleo MA, Boccardi M, Atripaldi L, Chignoli B, Maturo N, Rescigno C, Esposito V, Dell’Aversano R, Sangiovanni V, Punzi R (2020). COVID-19 among healthcare workers in a specialist infectious diseases setting in Naples, southern Italy: results of a cross-sectional surveillance study. J Hosp Infect.

[30] Di Ciaula A, Palmieri VO, Migliore G, Portincasa P, Group IMC (2020). COVID-19, internists and resilience: the north-South Italy outbreak. Eur J Clin Investig.

[31] ISTAT, Italian population. http://dati.istat.it/Index.aspx?DataSetCode=DCIS_POPRES1. Accessed on Mar 2021.

[32] Epicentro. Portale di epidemiologia per gli operatori sanitari. https://www.epicentro.iss.it/coronavirus/bollettino/Bollettino-sorveglianza-integrata-COVID-19_26-maggio-2020.pdf. Accessed on Dec 2020.

[33] regioni RSr. 2020. https://github.com/pcm-dpc/COVID-19/blob/master/schede-riepilogative/regioni/dpc-covid19-ita-scheda-regioni-20200531.pdf. Accessed on 4 June 2020.

[34] Gregori D, Azzolina D, Lanera C, Prosepe I, Destro N, Lorenzoni G, Berchialla P (2020). A first estimation of the impact of public health actions against COVID-19 in Veneto (Italy). J Epidemiol Community Health.

[35] Lavezzo E, Franchin E, Ciavarella C, Cuomo-Dannenburg G, Barzon L, Del Vecchio C (2021). Author correction: suppression of a SARS-CoV-2 outbreak in the Italian municipality of Vo. Nature..

[36] Hick JL, Christian MD, Sprung CL, European Society of Intensive Care Medicine’s Task Force for intensive care unit triage during an influenza epidemic or mass d. Chapter 2 (2010). Surge capacity and infrastructure considerations for mass critical care. Recommendations and standard operating procedures for intensive care unit and hospital preparations for an influenza epidemic or mass disaster. Intensive Care Med.

[37] Buoro S, Di Marco F, Rizzi M, Fabretti F, Lorini FL, Cesa S (2020). Papa Giovanni XXIII Bergamo Hospital at the time of the COVID-19 outbreak: letter from the warfront. Int J Lab Hematol.

[38] Grasselli G, Pesenti A, Cecconi M (2020). Critical care utilization for the COVID-19 outbreak in Lombardy, Italy: early experience and forecast during an emergency response. JAMA..

[39] Alzunitan MA, Perencevich EN, Edmond MB (2021). Assessing health care worker perceptions of face coverings during the COVID-19 pandemic. Am J Infect Control.

[40] (WHO) WHO. Rational use of personal protective equipment for coronavirus disease (COVID-19) and considerations during severe shortages: interim guidance, 6 April 2020. World Health Organization; 2020. https://apps.who.int/iris/handle/10665/331695. Accessed 7 Mar 2021. License: CC BY-NC-SA 3.0 IGO

[41] (WHO) WHO (2020). Novel Coronavirus (2019-nCoV).

[42] European Centre for Disease Prevention and Control (ECDC). Infection Prevention and Control For the Care of Patients With 2019-nCoV in Healthcare Settings. Available from: https://www.ecdc.europa.eu/sites/default/files/documents/nove-coronavirus-infection-prevention-control-patients-healthcare-settings.pdf. Accessed on 27 Mar 2020.

[43] Center for Disease Control and Prevention (CDC). Interim Healthcare Infection Prevention and Control Recommendations For Patients Under Investigation for 2019 Novel Coronavirus January 2020. Available from: https://www.cdc.gov/coronavirus/2019-nCoV/infection-control.html. Accessed on 27 Mar 2020.

[44] Kluytmans-van den Bergh MFQ, Buiting AGM, Pas SD, Bentvelsen RG, van den Bijllaardt W, van Oudheusden AJG (2020). Prevalence and clinical presentation of health care workers with symptoms of coronavirus disease 2019 in 2 Dutch hospitals during an early phase of the pandemic. JAMA Netw Open.

[45] Onder G, Rezza G, Brusaferro S (2020). Case-fatality rate and characteristics of patients dying in relation to COVID-19 in Italy. JAMA..

[46] Chen PZ, Bobrovitz N, Premji Z, Koopmans M, Fisman DN, Gu FX. Heterogeneity in transmissibility and shedding SARS-CoV-2 via droplets and aerosols. Elife. 2021;10. 10.7554/eLife.65774 PubMed PMID: 33861198. Epub 2021/04/17. 10.7554/eLife.65774 PMC8139838 33861198

[47] WHO Coronavirus disease (COVID-19): How is it transmitted? https://www.who.int/news-room/q-a-detail/coronavirus-disease-covid-19-how-is-it-transmitted. Accessed on May 2021.

[48] Troosters T, Tabin N, Langer D, Burtin C, Chatwin M, Clini EM, Emtner M, Gosselink R, Grant K, Inal-Ince D, Lewko A, Main E, Mitchell S, Niculescu A, Oberwaldner B, Pitta F (2019). Introduction of the harmonised respiratory physiotherapy curriculum. Breathe (Sheff).

[49] Nicola M, Sohrabi C, Mathew G, Kerwan A, Al-Jabir A, Griffin M (2020). Health policy and leadership models during the COVID-19 pandemic: a review. Int J Surg.

[50] Yau B, Vijh R, Prairie J, McKee G, Schwandt M. Lived experiences of frontline workers and leaders during COVID-19 outbreaks in long-term care: a qualitative study. Am J Infect Control. 2021; PubMed PMID: 33762181. Pubmed Central PMCID: PMC7981788. Epub 2021/03/26. eng. 10.1016/j.ajic.2021.03.006 PMC7981788 33762181

[51] Rizzo C, Campagna I, Pandolfi E, Croci I, Russo L, Ciampini S, et al. Knowledge and perception of COVID-19 pandemic during the first wave (Feb-May 2020): a cross-sectional study among Italian healthcare workers. Int J Environ Res Public Health. 2021;18(7). 10.3390/ijerph18073767 PubMed PMID: 33916577. Pubmed Central PMCID: PMC8038455. Epub 2021/05/01. eng. 10.3390/ijerph18073767 PMC8038455 33916577

