# Supplementary material for: Prevalence and associated factors of COVID-19 across Italian regions: a secondary analysis from a national survey on physiotherapists
Source: Arch Physiother. 2021 Dec 17;11:30. doi: 10.1186/s40945-021-00125-y (PMC8677342; doi:10.1186/s40945-021-00125-y)
Supplement: Supplementary file 2 — Additional file 2. Questionnaire. [file 40945_2021_125_MOESM2_ESM.docx]

# Additional File 2. Questionnaire

**INFORMED CONSENT**

1. Do you agree to participate in the study and treatment of your data?

- Yes
- No

**SECTION 1. DEMOGRAPHIC CHARACTERISTICS**

*In the following section you will be asked (MANDATORY) questions aimed at identifying different social and demographic groups*

1. What is your gender?

- Female
- Male

1. What is your age?

**SECTION 2. PERSONAL RISK OF EXPOSURE**

*In the following section you will be asked (MANDATORY) questions aimed at identifying personal variables*

1. Where do you live? (Please enter your 5-digit zip code)
2. Smoking behaviour

- Current smoker (more than 5 cigarettes/day)
- Current smoker (less than 5 cigarettes/day)
- Former smoker
- Never smoker

1. What is your weight? (i.e., 60 kg)
2. What is your height? (i.e., 170 cm)
3. Have you had your flu vaccination in the past 12 months?

- Yes
- No

1. How much time did you usually spend doing physical activity (before the emergency)?

- $\geq$ 150 min/week
- $\leq$ 150 min/week
- None

1. For which conditions have you been diagnosed, treated, medicated and/or monitored?

- Pulmonary diseases (i.e. asthma, chronic obstructive pulmonary disease)
- Cardiac diseases (i.e. coronary heart disease, atrial fibrillation)
- Hypertension
- Kidney diseases
- Immune system disorders (i.e. allergies, thyroiditis)
- Rheumatic diseases (i.e. rheumatoid arthritis, psoriasis)
- Oncologic conditions
- Metabolic diseases (i.e. diabetes, obesity, gout)
- Depression/anxiety
- Pregnancy
- Other conditions (i.e. surgery)
- None

1. Are you living with someone who might have coronavirus or has COVID-19 symptoms?

- Yes
- No

1. If yes, were/are you able to stay home, separate from the other persons living in the same household and using a separate bathroom? *

- Yes
- No

**SECTION 3. WORK-RELATED RISK OF EXPOSURE**

*In the following section you will be asked (MANDATORY) questions aimed at identifying risk factors in the workplace*

1. Where do you work? (Please enter your 5-digit zip code)
2. Currently, in what professional field do you mainly work?

- Orthopedic-Musculoskeletal
- Neurologic
- Oncologic
- Cardio-respiratory
- Urogynecologic
- Geriatric
- Pediatric
- Mixed

1. Select the facility you are working in:

- Private/Public hospital
- Residential Care Home
- Private setting
- Private/public rehabilitation clinics
- Home
- More (i.e. residential care home + private setting)

1. What is your current employment status?

- On duty at the workplace
- On duty on tele-work
- Not on duty (on vacation, leave, parental leave, layoff, sick leave)
- Not on duty (professional activity suspended by law or suspended due to COVID-19 outbreak)
- Not on duty (diagnosis of COVID-19)
- On quarantine (suspected COVID-19)

1. If you are on duty, have you been reallocated to a different unit (for example, from pediatric to respiratory area)? *

- Yes
- No

1. Did you change task job (for example, a physical therapist may be asked to support triage activities for COVID-19)? *

- Yes
- No

**SECTION 4. PREVALENCE (infection, signs and symptoms)**

*In the following section you will be asked (MANDATORY) questions aimed at identifying the prevalence of SARS-COV-2 infection.*

1. Do you have or think you might have COVID-19?

- Yes
- No

1. If you suspect or have contracted COVID-19, what signs and symptoms did you show (multiple choices allowed)*:

- Fever, with temperature above 37.5*C for at least three consecutive days
- Cough
- Fatigue, tiredness
- Headache
- Sore throat and/or sneezing
- Loss of smell
- Loss of taste
- Aches and pains
- Conjunctivitis/red eyes
- Respiratory issues
- Diagnosis of Pneumonia
- Difficulty breathing (shortness of breath at rest)
- Chest pain
- Tachycardia
- Diarrhea
- Other gastrointestinal problems
- No sign or symptoms/good health condition

1. If you presented with any signs or symptoms of COVID-19, in which month they appeared*:

- January
- February
- March
- April

1. If you answered yes to any of the above two questions, when you showed symptoms, at your workplace*:

- you had had contact with suspected/confirmed patients with COVID-19 (wearing Personal Protective Equipments)
- you had had contact with suspected/confirmed patients with COVID-19 (without Personal Protective Equipments)
- you had not had any known or confirmed contact with suspected/confirmed patients with COVID-19

1. Was a nasopharyngeal swab taken to test your exposure to SARS-COV-2?

- Yes, I tested positive
- Yes, I tested negative
- Yes, I do not know the result
- No, I have not been tested.

1. If you answered no, please explain why*:

- I did not present with symptoms requiring swab test to be taken
- The health surveillance services did not recommend it

1. Did you get any serological testing (blood drawn or rapid test) to assess your exposure to SARS-COV-2?

- Yes, I tested positive
- Yes, I tested negative
- Yes, I do not know the result
- No, I have not been tested

1. If you answered no, please explain why*:

- I did not present with symptoms requiring a serological test
- The health surveillance services did not recommend it

1. Have you been hospitalized because of COVID-19?

- Yes
- No

* adaptive questioning
