# Supplementary material for: Prevalence and associated factors of COVID-19 across Italian regions: a secondary analysis from a national survey on physiotherapists
Source: Arch Physiother. 2021 Dec 17;11:30. doi: 10.1186/s40945-021-00125-y (PMC8677342; doi:10.1186/s40945-021-00125-y)

# Additional File 3. Cluster analysis

## Table S1. Region response rate

| Region | Response Rate (%) |
| --- | --- |
| ABRUZZO | 16.6 |
| BASILICATA | 16 |
| SICILIA | 12.8 |
| CALABRIA | 14.8 |
| CAMPANIA | 13.4 |
| EMILIA-ROMAGNA | 33.6 |
| FRIULI-VENEZIA-GIULIA | 31.5 |
| LAZIO | 19.1 |
| LIGURIA | 25.2 |
| LOMBARDIA | 30.2 |
| MARCHE | 27.1 |
| MOLISE | 8.9 |
| PIEMONTE & VALLE D'AOSTA | 31.7 |
| PUGLIA | 13.7 |
| SARDEGNA | 19.1 |
| TOSCANA | 28.8 |
| TRENTINO-ALTO-ADIGE | 29.5 |
| UMBRIA | 28 |
| VENETO | 34.5 |

* Agglomerative nesting hierarchical clustering method used to identify groupings of regions based on response rates

## Figure S1. Region Dendrogram based on response rate


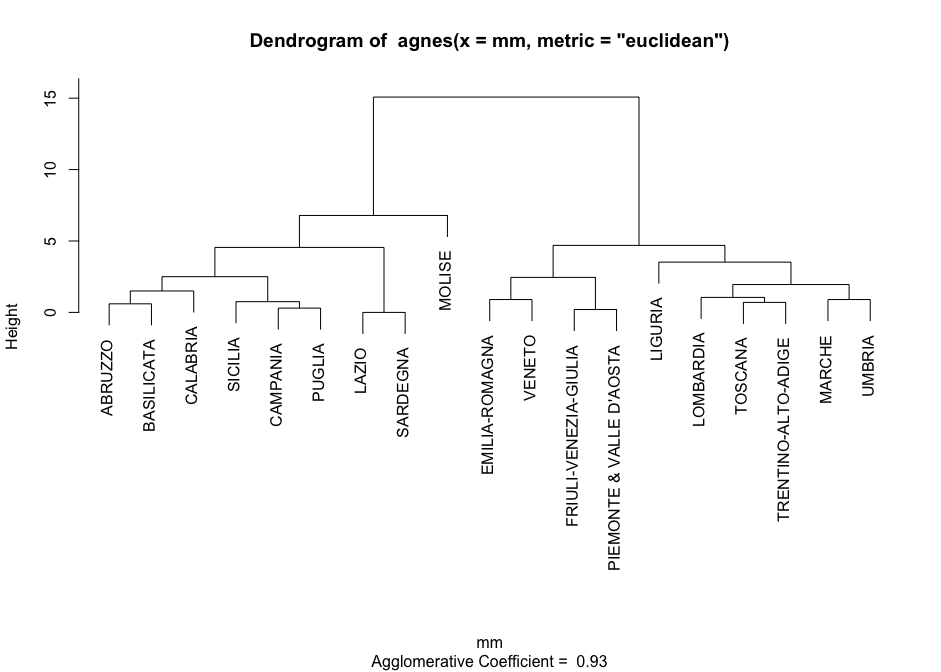


**Cluster 1**= Piedmont and Aosta Valley, Liguria, Lombardy, Veneto, Friuli-Venezia-Giulia, Trentino-Alto-Adige, Emilia-Romagna, Tuscany, Marche and Umbria; **Cluster 2**= Abruzzo, Lazio, Molise, Campania, Apulia, Basilicata, Calabria, Sicily and Sardinia

**Note:** The regions with the greatest statistical power (99% CI with type I error of 1%) were placed in Cluster 1 (the Northern and Central regions of Italy)

## Figure S2. Optimal number of clusters


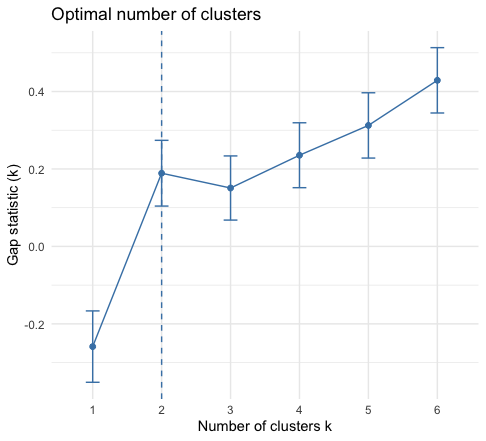

Supplement: Supplementary file 3 — Additional file 3. Cluster analysis. [file 40945_2021_125_MOESM3_ESM.docx]
