# Supplementary material for: Prevalence and associated factors of COVID-19 across Italian regions: a secondary analysis from a national survey on physiotherapists
Source: Arch Physiother. 2021 Dec 17;11:30. doi: 10.1186/s40945-021-00125-y (PMC8677342; doi:10.1186/s40945-021-00125-y)
Supplement: Supplementary file 4 — Additional file 4. Additional analyses Prevalence of COVID-19 in all regions. [file 40945_2021_125_MOESM4_ESM.docx]

# Additional File 4. Additional analyses Prevalence of COVID-19 in all regions

## Figure S1. Prevalence of NPS test provided per n° of PT respondents in each region


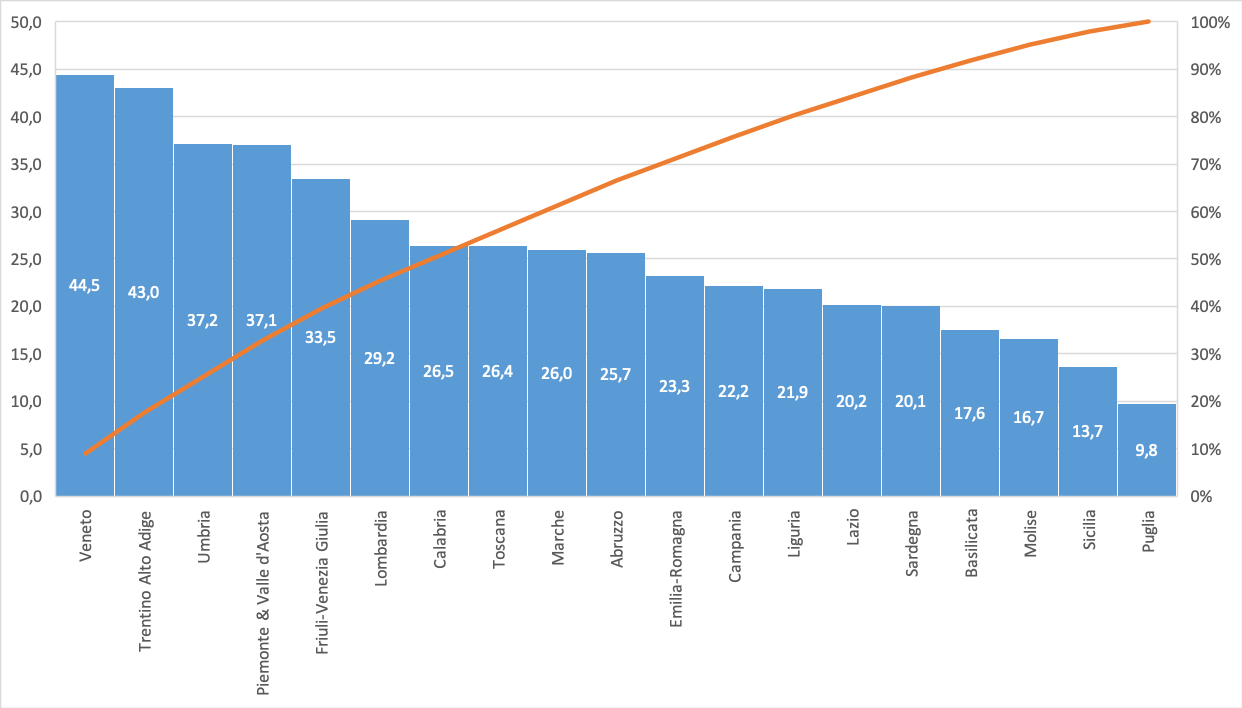


## Figure S2. Prevalence of positive COVID-19 cases, by NPS test, per n° of respondents in each region
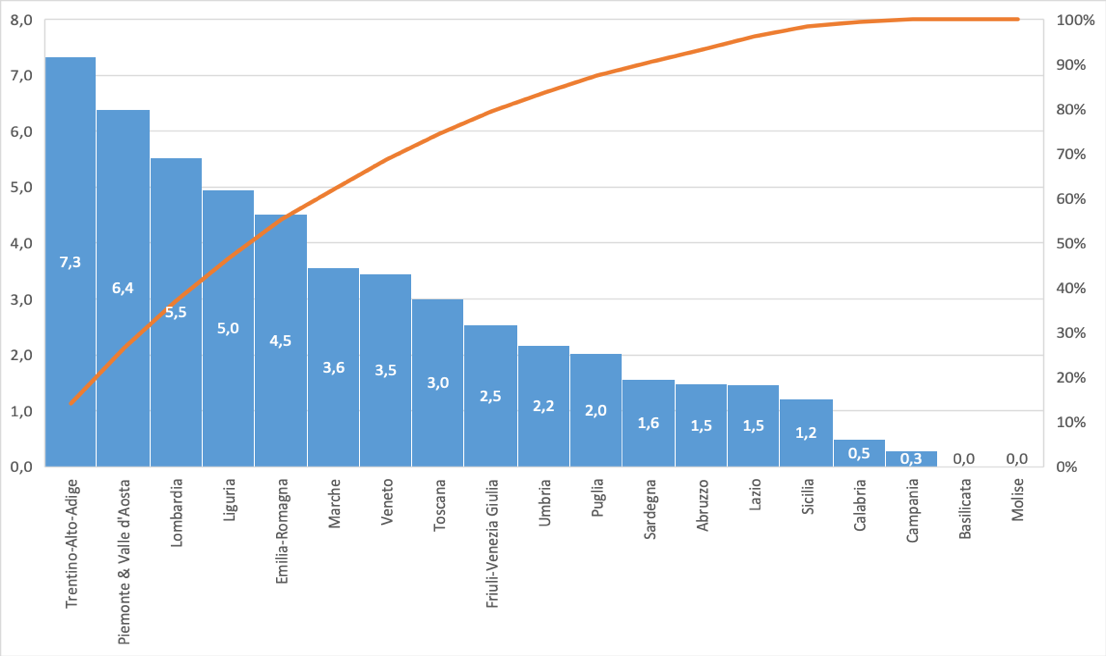


## Figure S3. Prevalence of positive COVID-19 cases on NPS test per n° of NPSs underwent in each region
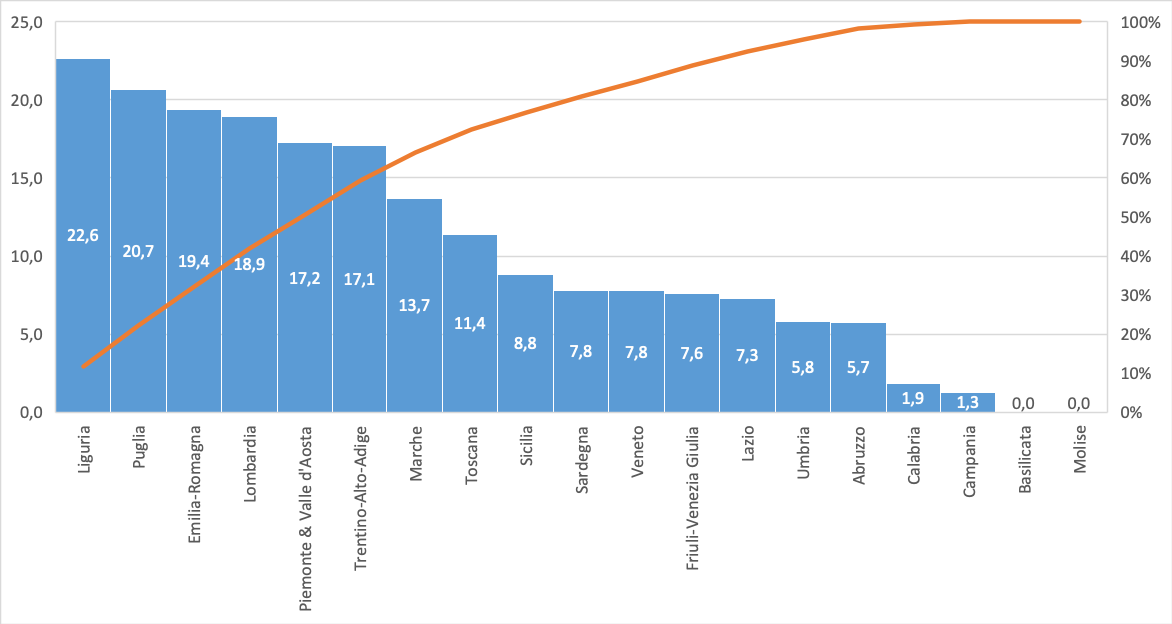


Among the total number of NPSs performed per each region, we found that Liguria had the most prevalent cases (22.6%), followed by Puglia (20.7%) and Emilia Romagna (19.4%).
