# Supplementary material for: Prevalence and associated factors of COVID-19 across Italian regions: a secondary analysis from a national survey on physiotherapists
Source: Arch Physiother. 2021 Dec 17;11:30. doi: 10.1186/s40945-021-00125-y (PMC8677342; doi:10.1186/s40945-021-00125-y)
Supplement: Supplementary file 5 — Additional file 5. Additional analyses symptoms of COVID-19 in all regions. [file 40945_2021_125_MOESM5_ESM.docx]

# Additional File 5. Additional analyses symptoms of COVID-19 in all regions

## Table S1. Prevalence of symptoms stratified by NPS test results for each cluster

| **SYMPTOMS LINKED TO KNOWN OR UNKNOWN SWAB RESULTS** | **POSITIVE NPS** | | **NEGATIVE NPS** | | **RESULT UNKNOWN** | | **NOT PERFORMED** | |
| --- | --- | --- | --- | --- | --- | --- | --- | --- |
|  | **Cluster 1** | **Cluster 2** | **Cluster 1** | **Cluster 2** | **Cluster 1** | **Cluster 2** | **Cluster 1** | **Cluster 2** |
| **N (%)** | 475 (100.0) | 55 (100.0) | 2549 (100.0) | 692 (100.0) | 189 | 692 (100.0) | 6385 | 692 (100.0) |
| **LOSS OF TASTE** | 273 (57.5) | 36 (65.5) | 129 (5.1) | 8 (1.2) | 24 (12.7) | 8 (1.2) | 219 (3.4) | 8 (1.2) |
| **LOSS OF SMELL** | 305 (64.2) | 37 (67.3) | 124 (4.9) | 6 (0.9) | 25 (13.2) | 6 (0.9) | 213 (3.3) | 6 (0.9) |
| **OTHER GASTROINTESTINAL PROBLEMS** | 79 (16.6) | 26 (47.3) | 51 (2.0) | 4 (0.6) | 8 (4.2) | 4 (0.6) | 86 (1.3) | 4 (0.6) |
| **CONJUNCTIVITIS/RED EYES** | 103 (21.7) | 28 (50.9) | 57 (2.2) | 2 (0.3) | 8 (4.2) | 2 (0.3) | 71 (1.1) | 2 (0.3) |
| **DIARRHEA** | 177 (37.3) | 33 (60.0) | 99 (3.9) | 9 (1.3) | 15 (7.9) | 9 (1.3) | 180 (2.8) | 9 (1.3) |
| **DIFFICULTY BREATHING (SHORTNESS OF BREATH AT REST)** | 113 (23.8) | 31 (56.4) | 47 (1.8) | 1 (0.1) | 9 (4.8) | 1 (0.1) | 79 (1.2) | 1 (0.1) |
| **CHEST PAIN** | 116 (24.4) | 33 (60.0) | 55 (2.2) | 3 (0.4) | 11 (5.8) | 3 (0.4) | 103 (1.6) | 3 (0.4) |
| **ACHES AND PAINS** | 283 (59.6) | 39 (70.9) | 152 (6.0) | 8 (1.2) | 31 (16.4) | 8 (1.2) | 278 (4.4) | 8 (1.2) |
| **FATIGUE, TIREDNESS** | 321 (67.6) | 45 (81.8) | 218 (8.6) | 8 (1.2) | 36 (19.0) | 8 (1.2) | 392 (6.1) | 8 (1.2) |
| **FEVER > 37.5°C FOR AT LEAST 3 DAYS** | 223 (46.9) | 36 (65.5) | 125 (4.9) | 5 (0.7) | 24 (12.7) | 5 (0.7) | 211 (3.3) | 5 (0.7) |
| **SORE THROAT AND/OR SNEEZING** | 207 (43.6) | 34 (61.8) | 138 (5.4) | 4 (0.6) | 18 (9.5) | 4 (0.6) | 246 (3.9) | 4 (0.6) |
| **HEADACHE** | 234 (49.3) | 37 (67.3) | 157 (6.2) | 7 (1.0) | 20 (10.6) | 7 (1.0) | 235 (3.7) | 7 (1.0) |
| **RESPIRATORY ISSUES** | 86 (18.1) | 27 (49.1) | 25 (1.0) | 1 (0.1) | 3 (1.6) | 1 (0.1) | 44 (0.7) | 1 (0.1) |
| **TACHYCARDIA** | 91 (19.2) | 31 (56.4) | 50 (2.0) | 2 (0.3) | 6 (3.2) | 2 (0.3) | 53 (0.8) | 2 (0.3) |
| **COUGH** | 226 (47.6) | 36 (65.5) | 130 (5.1) | 4 (0.6) | 18 (9.5) | 4 (0.6) | 251 (3.9) | 4 (0.6) |
| **NO SYMPTOMS** | 49 (10.3) | 5 (9.1) | 2232 (87.6) | 673 (97.3) | 143 (75.7) | 673 (97.3) | 5809 (91.0) | 673 (97.3) |

**Legend:** Cluster 1= Piedmont and Aosta Valley, Liguria, Lombardy, Veneto, Friuli-Venezia-Giulia, Trentino-Alto-Adige, Emilia-Romagna, Tuscany, Marche and Umbria; Cluster 2= Abruzzo, Lazio, Molise, Campania, Apulia, Basilicata, Calabria, Sicily and Sardinia

## Figure S1. Sign and symptoms in clusters stratified by positive NPS results

**
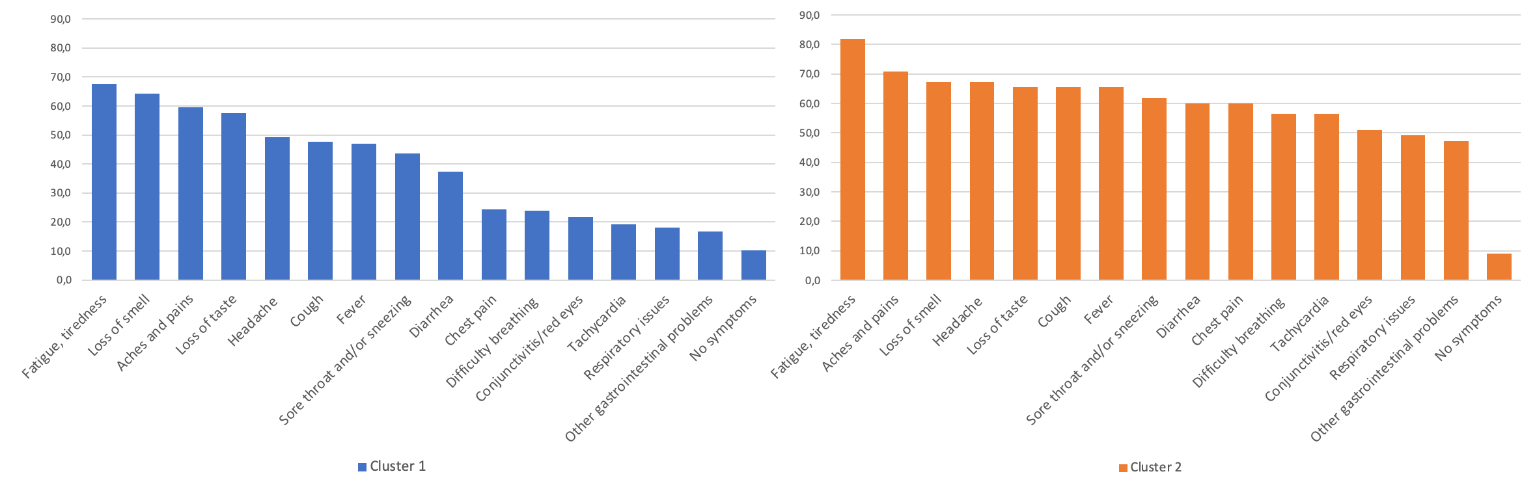
**

**Legend:** Cluster 1= Piedmont and Aosta Valley, Liguria, Lombardy, Veneto, Friuli-Venezia-Giulia, Trentino-Alto-Adige, Emilia-Romagna, Tuscany, Marche and Umbria; Cluster 2= Abruzzo, Lazio, Molise, Campania, Apulia, Basilicata, Calabria, Sicily and Sardinia

In Cluster 1 (n=475), the most prevalent symptoms were fatigue and tiredness (67.6%), loss of smell (64.2%), aches and pains (59.6%), loss of taste (57.5%) and headache (49.3%), whereas in Cluster 2 (n=55), fatigue and tiredness (81.8%), aches and pain (70.9%), loss of smell (67.3%), headache (67.3%) and loss of taste (65.5%) were the most common symptoms. No symptoms were reported by 10.3% and 9.1% of the respondents in Clusters 1 and 2, respectively.
