# Supplementary material for: Prevalence and associated factors of COVID-19 across Italian regions: a secondary analysis from a national survey on physiotherapists
Source: Arch Physiother. 2021 Dec 17;11:30. doi: 10.1186/s40945-021-00125-y (PMC8677342; doi:10.1186/s40945-021-00125-y)
Supplement: Supplementary file 6 — Additional file 6. Additional analyses risk exposure of COVID-19 in all regions. [file 40945_2021_125_MOESM6_ESM.docx]

# Additional File 6. Additional analyses risk exposure of COVID-19 in all regions

## Table S1. Characteristics of PTs with missing COVID test results in cluster 1 and cluster 2 (n=936).

|  | **Cluster 1** | **Cluster 2** |
| --- | --- | --- |
| **Sex (male)** | 200 (34.9%) | 130 (35.8%) |
| **Age** | 42.8 (12.7) | 42.7 (12.4) |
| **Comorbidities (at least 1)** | 81 (14.1%) | 50 (13.8%) |
| **Field** |  |  |
| Cardio-Thorax | - | 1 (0.3%) |
| Geriatric | 8 (1.4%) | - |
| Neurologic | 1 (0.2%) | 3 (0.8%) |
| Orthopedic-Musculoskeletal | 19 (3.3%) | 6 (1.6%) |
| Pediatric | 1 (0.2%) | - |
| Mixed | 26(4.5%) | 12 (3.3%) |
| Urogynecologic and Oncologic | - | - |
| *missing* | 518 (90.4%) | 341 (93.9%) |
| **Workplace** |  |  |
| Private/public hospital as reference | 13 (2.3%) | 5 (1,4%) |
| Residential Care Home | 8 (1.4%) | - |
| Private/public rehabilitation clinics | 6 (1,1%) | 6 (1.7%) |
| Home | 4 (0.7%) | 4 (1.1%) |
| Private setting | 15 (2.6%) | 4 (1.1%) |
| Mixed | 9 (1.6%) | 3 (0.8%) |
| *missing* | 518 (90.4%) | 341 (93.9%) |

**Legend:** Cluster 1= Piedmont and Aosta Valley, Liguria, Lombardy, Veneto, Friuli-Venezia-Giulia, Trentino-Alto-Adige, Emilia-Romagna, Tuscany, Marche and Umbria; Cluster 2= Abruzzo, Lazio, Molise, Campania, Apulia, Basilicata, Calabria, Sicily and Sardinia
